# Barriers and facilitators to engaging with a digital self‐management programme for painful distal upper limb musculoskeletal disorders: A qualitative exploratory study

**DOI:** 10.1111/hex.14056

**Published:** 2024-06-10

**Authors:** Samantha J. Mason, Lucy M. Brading, Kathleen Kane, Philip G. Conaghan, Sarah R. Kingsbury, Gretl A. McHugh

**Affiliations:** ^1^ Leeds Institute of Rheumatic and Musculoskeletal Medicine University of Leeds Leeds UK; ^2^ Bone Cancer Research Trust Leeds UK; ^3^ NIHR Leeds Biomedical Research Centre Leeds UK; ^4^ School of Healthcare University of Leeds Leeds UK

**Keywords:** digital engagement, distal upper limb, musculoskeletal, patient and public involvement and engagement, qualitative, rehabilitation, self‐management

## Abstract

**Introduction:**

People living with a painful distal upper limb musculoskeletal disorder (DUL‐MSD) often experience pain, difficulty in doing everyday tasks and a reduced quality of life. Currently, there are challenges in the treatment of DUL‐MSDs, highlighting the need to develop innovative approaches to rehabilitation. A potential solution is to develop and implement a digital self‐management rehabilitation programme focussing on optimising recovery, improving function and reducing pain. Before developing this programme, we aimed to identify the barriers and facilitators to using a digital health intervention (DHI) for self‐management of DUL‐MSDs.

**Objective:**

This study aimed to investigate the potential barriers and facilitators to using a DHI with people living with DUL‐MSDs and healthcare professionals (HCPs).

**Methods:**

A qualitative exploratory study was carried out with purposely selected participants consisting of 15 participants with DUL‐MSDs and 13 HCPs. Three focus groups (FGs) and four semistructured interviews with DUL‐MSD participants and semistructured interviews with 13 HCPs were conducted. FGs and interviews were digitally recorded, transcribed and analysed using reflexive thematic analysis.

**Results:**

To address challenges in the care and management of DUL‐MSDs, both HCPs and people living with a DUL‐MSD welcomed the development of a DHI. This study identified several barriers and facilitators that would influence engagement with a digital intervention. Findings suggest that in developing a DHI, attention needs to be paid to digital design features, usability, tailoring, personalisation and consideration of how well usual care could be replicated digitally without direct HCP involvement.

**Conclusion:**

The identified digital design features of importance to participants will inform the design of a digital self‐management rehabilitation programme for people living with DUL‐MSDs. Addressing the barriers and facilitators to engagement with a DHI is essential in ensuring its relevance and acceptability to those who will use it.

**Patient or Public Contribution:**

Patient and Public Involvement and Engagement (PPIE) was integral throughout the study. PPIE members contributed to the development and planning of this study, checked and confirmed the relevance of the findings and are involved in the dissemination plans.

## 1 INTRODUCTION

Experiencing pain in the distal upper limb (DUL) region is common and often due to a musculoskeletal disorder (MSD).^1^ Common DUL‐MSDs include hand and thumb osteoarthritis (OA), epicondylitis, tenosynovitis and carpal tunnel syndrome. Some people also experience pain in the forearm that is undiagnosed and is described as having ‘non‐specific arm pain’, previously known as repetitive strain injury.^2^ The prevalence of DUL‐MSDs varies with the specific disorder, with hand OA affecting 27% of the population,^3^ carpal tunnel syndrome affecting between 1% and 16%^4^, ^5^ and tenosynovitis and epicondylitis affecting between 1% and 3%.^6^, ^7^ MSDs account for 21% of years living with a disability, a measure that considers the prevalence of the disease and how disabling the disease is.^8^ Many DUL‐MSDs are common among working‐age adults, impacting the workforce and having economic consequences for both employees and employers.^9^, ^10^ Work‐related MSDs are also an issue and certain occupations are at an increased risk of developing a DUL‐MSD.^11^, ^12^

The effects of living with a DUL‐MSD can be immense. People living with a DUL‐MSD may experience persistent pain that impacts their quality of life, increasing levels of disability and difficulty in carrying out activities of daily living.^13^, ^14^, ^15^, ^16^ For example, people with hand OA experience functional limitations and have expressed concern over their inability to carry out ‘normal’ daily tasks, with related psychological effects.^17^

Healthcare professionals (HCPs) find the treatment and management of DUL‐MSDs challenging. This is partly due to the lack of evidence base about the effectiveness of treatment options.^18^ There are some guidelines for the management of specific disorders such as carpal tunnel syndrome^4^ and EULAR recommendations for the management of hand OA.^19^ Some guidance on occupational management of upper limb disorders has also been published.^20^, ^21^

Specific types of exercises such as grip‐strengthening exercises are often recommended for the improvement of DUL‐MSDs.^22^ However, exercise rehabilitation requires completion over several weeks and there are difficulties in sustaining adherence to exercise programmes,^23^ with a lack of evidence about intensity and dosage of exercise programmes.^22^ Exercise may be optimised with guided self‐management support.^24^

Discussions with a diverse Patient and Public Involvement and Engagement (PPIE) group living with DUL‐MSDs revealed dissatisfaction with current treatment and management. They wanted more support and continued exercise therapies, which were individualised for their specific DUL‐MSD while taking into account their capabilities.

To address challenges and deficits in the management of these painful and disabling conditions, self‐management support for people living with DUL‐MSDs is needed. With the National Health Service (NHS) focussed on digital transformation,^25^ developing a digital health self‐management rehabilitation programme for people with DUL‐MSDs was considered as a potential solution. Evidence supports the use of digital health interventions (DHIs) for reducing pain, improving physical functioning and self‐management of painful musculoskeletal conditions^26^, ^27^ and improving exercise adherence.^28^

The aim of this study was to investigate the potential barriers and facilitators to using a DHI with people living with DUL‐MSDs and HCPs. This study formed the initial phase of a larger programme of research and these findings will inform the next stage of this programme of research, development of a digital self‐management rehabilitation programme.

## 2 METHODS

This study was part of the intervention planning phase and designed as an exploratory qualitative study using a theory‐based approach.^29^, ^30^, ^31^ This work was undertaken to ensure that the DHI is relevant, accessible and useable for individuals with DUL‐MSDs. The study's reporting is guided by the Consolidated Criteria for Reporting Qualitative Research checklist.^32^

### 2.1 PPIE

Ten PPIE members contributed to the study, all of whom live with DUL‐MSDs. The group contributed throughout the project and reviewed participant‐facing information, piloted topic guide questions and assisted with interpretation of findings and dissemination.

### 2.2 Participants and recruitment

Participants with lived experience of DUL‐MSDs were recruited via social media; advertising through third sector, educational organisations and NHS trusts; and patient mail‐outs co‐ordinated by rheumatology and musculoskeletal services within an NHS Hospital Trust and a community NHS Trust. HCPs were recruited via social media and advertising through professional networks.

Individuals with DUL‐MSDs were eligible for inclusion if they were at least 18 years old; able to communicate in English; and had a medical diagnosis or self‐diagnosis of a DUL‐MSD that included hand and thumb OA, epicondylitis (tennis elbow or golfer's elbow), tendinitis/tenosynovitis, carpal tunnel syndrome or nonspecific arm pain. HCPs were eligible for inclusion if they had at least three years' experience of working with these conditions.

Initially, the study set out to apply purposive sampling^33^, ^34^ for recruitment of people with DUL‐MSDs and criteria included condition type, sex, ethnicity and confidence using the internet. However, challenges to recruitment of sufficient participants within the available study timeframe meant that a more pragmatic approach was applied: recruiting based on interest and availability rather than meeting all purposive criteria. HCPs were purposively sampled based on profession, enabling different perspectives in the care and management of DUL‐MSDs to be explored.

From our recruitment strategies, we received expressions of interest from potential participants and invited 63 people living with DUL‐MSDs and 37 HCPs to take part in the study, providing them with a participant information sheet. Of these, 13 HCPs and 23 people with DUL‐MSDs consented. Eight people who consented were not able to ultimately take part. Data saturation, when no new themes were identified, was used to guide our sample size.^35^, ^36^ Data saturation was achieved following interviews and focus groups (FGs) with 15 people living with DUL‐MSDs (three FGs with 11 participants and four semistructured virtual interviews) and 13 semistructured HCP interviews.

### 2.3 Data collection

Before participating in a FG or interview, participants completed an electronic consent form and online questionnaire. The questionnaire asked about sociodemographic characteristics, type of DUL‐MSD, usual care, level of pain using a numerical rating scale^37^, ^38^ and confidence using the internet, self‐rated on a five‐point scale.^39^ HCPs completed questions relating to their professional role and experience. Interviews and FGs were arranged at a time to suit the participants. DUL‐MSD participants who were unable to or did not wish to attend a FG were offered one‐to‐one interviews to not exclude interested participants.

Topic guides for interviews and FGs were developed based on existing literature^40^, ^41^ and engagement with the PPIE group and structured using the capabilities, opportunities, motivation, behavior (COM‐B) model.^42^ The topic guide for participants with DUL‐MSDs focussed upon

1. Care and management of DUL‐MSDs;
2. experience of exercises for DUL‐MSDs;
3. use of websites for self‐management of DUL‐MSD and other health conditions; and
4. barriers/facilitators to using a DHI to support self‐management of DUL‐MSDs.

Examples of digital features, referred to as trigger materials, were developed and shown to participants (Supporting Information S1: File 1). Participants discussed the usefulness of these design features to help self‐manage their DUL‐MSD.

The topic guide for HCPs focussed upon

1. Patient care and management (including improvements);
2. information provision (including availability, deficits);
3. perceived barriers to self‐management advice/support;
4. views about a DHI to support self‐management of DUL‐ MSDs (exploring: use, accessibility, implementation challenges); and
5. design and content of a DHI (exploring exercises, motivation/confidence levels, skill levels).

One FG with participants with DUL‐MSDs was conducted in person at a university location, while a further two FGs were conducted by videoconferencing. Four participants who were unable to attend FGs participated in individual virtual interviews. Interviews and FGs took place between April and August 2022. FGs lasted between 75 and 78 min and interviews lasted between 39 and 76 min (mean 53 min). FGs were facilitated by two researchers (L. M. B./K. K.).

HCP interviews were conducted by L. M. B./K. K. using videoconferencing, with one conducted by telephone due to connectivity issues. HCPs' interviews took place between March and September 2022 and lasted between 27 and 81 min (mean 54 min).

All FGs and interviews were digitally recorded using an encrypted audio‐recorder and/or the record function of Microsoft teams/Zoom. Recordings were transcribed verbatim by a professional transcribing company.

### 2.4 Analysis

Reflexive thematic analysis was carried out to enable full data exploration and familiarisation.^43^, ^44^ Within this, an inductive approach was used, which allowed researchers to identify themes from the data and use their judgement when deciding upon themes. Data from HCPs and participants with DUL‐MSDs were analysed together. Themes were identified and agreed by three researchers with qualitative research experience, based on insights and the ability of the themes to capture important barriers and facilitators. Themes were mostly identified at the semantic (descriptive) level, before undertaking further work to understand the underlying meaning of themes. Multiple strategies were used to enhance trustworthiness and rigour, including addressing trustworthiness criteria proposed by Lincoln and Guba^45^ (Table 1).

**Table 1:** Trustworthiness criteria and strategies used to address them (adapted from Lincoln and Guba).^45^

Criterion	Strategies
Credibility	The topic guide was developed and tested with the PPIE group and was followed to reduce researcher bias.
	Focus groups were used to encourage participants to freely express their perspectives, but individual interviews were offered to not exclude participants who wanted to take part in the study.
	Rapport was established with all participants before the focus groups and interviews. Participants knew that the facilitators' role was to understand their lived experiences and opinions about what would encourage and discourage them from using a DHI.
	The findings were integrated with other data sources and work package findings during the subsequent phases of the project.
	The findings informed the DHI prototype and feedback on the DHI prototype will be obtained through mixed‐methods research.
Confirmability	Audio recordings were transcribed by a professional transcription company, and then verified by a member of the research team.
	Coding was inductive and focussed on manifest content.
	All three researchers involved in the data interpretation read the data transcripts.
	The lead researcher discussed the data analysis with the other research team members.
	The lead researcher used a reflexive approach, including analysing the data using reflexive thematic analysis and keeping all iterative versions of coding documents and documenting what updates had been made from previous versions.
	Quotes are provided to support the themes and subthemes.
Dependability	Detailed information is provided about the study procedures and no changes were made to the procedures during the study.
	Repeat interviews and focus groups were not conducted.
	An audit trail was maintained, including annotated anonymised transcripts and iterative versions of coding documents showing updated themes and subthemes.
Transferability	As member checking was not undertaken, the PPIE group helped to confirm the findings.
	Detailed information is provided about the study design, context and participants.
	Findings from the current study were further investigated as part of evidence‐based co‐design sessions with both people living with DUL‐MSDs and healthcare professionals.

Abbreviations: DHI, Digital Health Intervention; DUL‐MSDs, Distal Upper Limb Musculoskeletal Disorders; PPIE, Patient and Public Involvement and Engagement.

Transcripts were checked, verified and corrected where necessary by listening to the corresponding audio recording and anonymised to ensure that participant confidentiality was maintained and data were not identifiable. S. J. M., L. M. B. and G. A. M. read the transcripts for familiarisation. Patterns within the data were observed and noted. Initial codes were identified and data were organised into meaningful groups. QSR International NVivo software (Version 12) was used to facilitate data organisation. Following this, more inductive coding was undertaken, identifying issues of importance and key barriers and facilitators to using a DHI. Codes were iteratively developed and clustered into categories based on their similarities and themes/subthemes were generated. At this stage, mapping of the themes and subthemes was undertaken and updated throughout the analytical process. Themes and subthemes were reviewed, refined, defined and allocated names. These processes were primarily undertaken by S. J. M., who discussed the analysis with other researchers (L. M. B./G. A. M.) to sense‐check findings, confirm themes and content and agree on how to progress. An analytic narrative was written for each theme, subtheme and important components of the subthemes, which were supported by data extracts. Finally, data and themes were organised to provide the most coherent narrative and the meaning of themes was considered, beyond simplistic descriptions.

## 3 FINDINGS

### 3.1 Participants

Participants consisted of 13 women and two men with DUL‐MSDs, aged between 32 and 71 years. Participants had hand/thumb OA (*n* = 6), tendinitis/tenosynovitis (*n* = 3), epicondylitis (*n* = 2), carpal tunnel syndrome (*n* = 1) and multiple DUL‐MSDs (*n* = 3). All participants except one had experienced symptoms for more than a year. Additional information is provided in Table 2.

**Table 2:** Participant characteristics of people with distal upper limb musculoskeletal disorders.

	Number of participants (%) (*n* = 15)
Age (years)	
30–39	1 (7)
40‐49	1 (7)
50–59	8 (53)
60–69	4 (27)
70–79	1 (7)
Gender	
Female	13 (87)
Male	2 (13)
Confidence in using the Internet^a^	
Very confident	7 (47)
Fairly confident	8 (53)
Distal upper limb musculoskeletal disorder	
Hand/thumb osteoarthritis	6 (40)
Epicondylitis	2 (13)
Tendinitis/tenosynovitis	3 (20)
Carpal tunnel syndrome	1 (7)
Multiple conditions	3 (20)
Duration of symptoms	
Between 6 and 12 months	1 (7)
>1 year	14 (93)
Pain rating^b^	
0–3	1 (7)
4–7	9 (60)
8–10	5 (33)
Ethnicity	
White British	15 (100)
Highest educational qualification	
GCSE/O‐level (or equivalent)	4 (27)
A‐level (or equivalent)	1 (7)
Vocational qualification	5 (33)
Undergraduate degree	1 (7)
Postgraduate degree	4 (27)
Current employment status	
Employed full‐time	6 (40)
Employed part‐time	2 (13)
Self‐employed	2 (13)
Student/in education	1 (7)
Retired	3 (20)
Unable to work due to ill health	1 (7)

Abbreviation: GCSE/O, General Certificate of Secondary Education.

^a^ Measured using the five‐point scale adapted from Pearson et al.^39^

^b^ Measured using the 11‐point Pain Numeric Rating Scale from Jensen and McFarland^38^.

HCPs included general practitioners (*n* = 2), hand therapists (*n* = 2), occupational therapists (*n* = 3), physiotherapists (*n* = 2), first contact practitioner physiotherapists (*n* = 2), a rheumatologist (*n* = 1) and an orthopaedic surgeon (*n* = 1). The majority worked in secondary care (*n* = 8), with four HCPs from primary care and one HCP from a private care provider.

### 3.2 Thematic analysis overview

**Figure 1:**
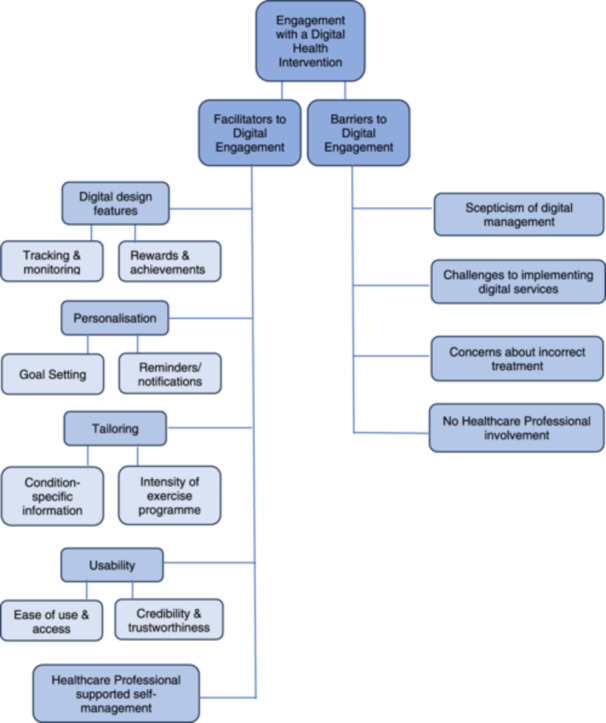
Engagement with a digital health intervention: Overview of themes and subthemes.

The results presented here focus on engagement with a DHI and themes were grouped according to whether they were facilitators or barriers to engagement. Five themes facilitated engagement and four themes were recognised as barriers to engagement (see Figure 1). Related subthemes were identified for facilitator themes, but not for barrier themes.

### 3.3 Facilitators to digital engagement

#### 3.3.1 Theme 1: Digital design features

Digital design features refer to specific components that would encourage users to engage with DHIs. Participants with DUL‐MSDs predominantly discussed two digital design features: ‘tracking and monitoring’ and ‘rewards and achievements’.

*Tracking and monitoring*: The ability to review data to track and monitor progress was the most frequently discussed design feature. Two elements were discussed by participants with DUL‐MSDs: how they wanted to track information and what they gained from this.

Participants enthused about being able to ‘tick things off’ to demonstrate completion of tasks and log activities. A key reason for tracking data was to review whether they were progressing as anticipated. They wanted to track exercise progress, pain levels, overall health and how they were feeling. Findings indicated that some of our participants used digital tools to track an array of health‐related metrics such as steps, heart rate, sleep and water intake.

A valued benefit of tracking data was comparing recorded information with how they physically felt. Several participants highlighted that it was reassuring to see how far they had come, especially when they thought that they were not progressing well. When discussing a physiotherapy‐based app recommended while receiving private healthcare, one FG participant said:

When you looked back it was sort of a good sort of memory of how you felt really a few weeks ago, so at the bit where you didn't see the physio every week it was just a good gauge to sort of see how, you might not feel like you're any better but when you look back you think ‘oh actually yes I was because I was scoring an 8 then, whereas this week I'm only scoring a 6 so the pain levels are coming down’. So it was useful for me just as a tracker to see how if it had been improving or if it was getting worse. (P5/FG2, Female‐54 years‐Epicondylitis)

*Rewards and achievements*: Rewards and achievements were regarded as incentives to use a DHI. The inclusion of a reward streak for daily use of the intervention and unlocking additional content through regular interaction were discussed. Unlocking additional exercises or articles were cited as rewards that may encourage engagement.

Perhaps if you had certain exercises that unlocked, as it got, you know as you progressed through your exercises you feel like you maybe start simply and it progresses in difficulty maybe that would be rewarding […] when you do something like where you are progressing the intensity of the exercises or you get additional ones added. (P13/Interview, Female‐32 years‐Tendinitis/Tenosynovitis)

#### 3.3.2 Theme 2: Personalisation

Most participants with DUL‐MSDs thought that users should be able to personalise and filter online content for their own individual needs. Personalised components included goal setting and reminders/notifications.

*Goal setting*: Goal setting is an important part of self‐management that allows people to manage their condition and achieve self‐directed outcomes. However, goal setting was not discussed in the same way by participants with DUL‐MSDs and HCPs. Using ‘SMART’ goals^46^ and action planning were discussed by HCPs, but not explicitly discussed by participants with DUL‐MSDs. While several understood the benefit of setting exercise goals and including this feature within a DHI, only one person currently had an exercise‐specific goal, which was the 10,000‐step goal that is preset by a smartwatch. Conversely, one participant avoided goal setting as they felt frustrated when they did not achieve their desired outcome.

I'm not so much a person who tends to set myself goals because then I get really mad at myself if I don't meet them. I like it to give me an idea of what to do and then I like to track whether I've done it or not. (P4/FG1, Female‐57 years‐Hand/Thumb OA)

In contrast, HCPs were more likely to talk about planning and meeting exercise‐related goals and the benefits. HCPs recognised goal setting as a positive activity to focus attention and considered the importance of individual circumstances when developing goals, so that goals were achievable.

Goal setting's a really good one because it focuses people's attention. (HCP05 Hand Therapist)

*Reminders/notifications*: Reminders and notifications were perceived as having two main purposes. One was to prompt users to engage with a DHI, to ensure compliance, and the other was to notify users of time points where they would typically expect to see improvements. Understanding a timeframe for improvements in their condition was a key piece of information that participants with DUL‐MSDs desired for expectation setting. Having this notification was important as it would enable users to seek HCP support if their condition had not improved. Notifications were viewed as potential motivators, acting as progress reminders and encouraging ongoing DHI use for maintaining or improving results.

Say for example like you've reached a month or something like that you might want something that can kind of say […] this is what you've achieved, you know you've done it this many times, you know the benefits of it again sort of like prompting people to do it by motivating them that way. (P13/Interview, Female‐32‐Tendinitis/Tenosynovitis)

#### 3.3.3 Theme 3: Tailoring

Participants with DUL‐MSDs referred to ‘tailoring’ in terms of customising the intervention for their own individual needs (which we defined as personalisation). Where participants with DUL‐MSDs discussed design features or content that addressed group requirements, rather than individual ones and could be grouped according to the user's condition or rate of progression through the programme, we categorised this as tailoring. The value of condition‐specific information and customising the intensity of the exercise programme were highlighted.

*Condition‐specific information*: Tailoring content according to condition was discussed by both FG 1 and HCPs. Condition‐specific information was preferred over generic information, which participants with DUL‐MSDs perceived as not applicable to them. Interesting and relevant content specific to their DUL‐MSD was preferred, while too much generic information was viewed as overwhelming, burdensome and would discourage use of a DHI.

I think something that was targeted at your own symptoms and your own things, I'm more likely to take notice of, whereas if it's just a generic thing, have you read this and have you read that? Well, most of what you read is irrelevant so then you lose interest. (P4/FG1, Female‐57 years‐Hand/Thumb OA)

*Intensity of exercise programme*: Both HCPs and participants with DUL‐MSDs recognised the benefits of customising the intervention to suit user requirements. Participants had positive impressions of apps that allowed them to choose the pace and intensity of exercise, based on their ability and pain levels. HCPs and participants with DUL‐MSDs expressed a desire for a customisable exercise programme that adapts or adds additional exercises as users progress, but where users can determine the pace and opt to do less strenuous exercises, as required.

On a day‐by‐day basis I can choose the intensity of the class, so if I wanted to do something that's a lot of core, a lot of like fat burn, you can do that, you can do a high intensity quite advanced level class, but actually if I've had a really bad night with pain, I've not slept, I can do like a beginners yoga class that day and I've still done my yoga, I've still done my exercise, I've still done my stretch, but I haven't damaged anything and I felt like I've achieved. (P6/FG2, Female‐50 years‐Hand/Thumb OA)

Have something that gets a little bit harder week‐on‐week and you have something that knows how to add the sets and reps up. (HCP01 Hand therapist)

#### 3.3.4 Theme 4: Usability

Usability of the intervention was a key consideration, with importance placed on ease of use and access. Improving the user experience was discussed, with the importance of a simple and convenient DHI containing credible, reliable and verified information emphasised throughout interviews and FGs.

*Ease of use and access*: Ease of use was recognised as vital in enabling a variety of users to engage with DHIs. While confidence using the internet was high amongst our participants, they highlighted that a simple, accessible app is essential for users who are not ‘tech savvy’. One FG participant mentioned that their experience using an exercise app during the Covid‐19 lockdowns had been so positive that they continued to use it instead of returning to traditional exercise classes.

I found that really useful and that's the one I'm still using now. That's the one that's probably stuck the longest because I felt like it was usable and, you know, it just worked for me. And even though yoga's now open again I've never gone back. (P6/FG2, Female‐50 years‐Hand/Thumb OA)

Participants highlighted the importance of an online intervention that could be accessed when needed, as it would be faster than waiting for usual care appointments. HCPs considered how a functional DHI could be more beneficial to certain patients than usual care and potentially save clinical time.

Having something else that can maybe at least do some of that, if a GP doesn't have time in their own 10‐minute consultation, if they have something else that can help the patients which saves them having to be referred which in turn will then improve waiting times. (HCP02 Rheumatologist)

*Credibility and trustworthiness*: DHIs were considered by participants with DUL‐MSDs to have high usability if they were perceived as credible, safe and secure. Information endorsed by reputable organisations such as foundations, charities or the National Health Service (NHS) was perceived as highly credible. The NHS was perceived as the ‘gold standard’ in terms of trustworthiness. Comparisons were made between NHS apps and other apps, with concerns raised about potential misuse of personal data in non‐NHS apps. HCPs identified that patients may have concerns about who could access their data. An exemplar quote was:

I generally steer very firmly towards anything that's NHS sponsored or by an NHS representative to do with those sorts of things […] anyone can create an app and anyone can put content on YouTube or they can share it on TikTok. (P9/FG3, Male‐40 years‐Epicondylitis)

Participants wanted access to clear, reliable, verified information that sets expectations, reassures and empowers. Examples given by both participants and HCPs included explanations for why specific exercises were recommended, timeframes for expected results, reassuring information if setbacks occur, pacing guidance, underlying causes of pain and pain management strategies.

If it tells you what and when you might be expected to see results as well, like a timeframe. (P2/FG1, Female‐55 years‐Tendinitis/Tenosynovitis)

Guidance about how often they should do the exercises per whatever condition that they've got and maybe some information on sort of joint education and pacing. (HCP06 Occupational Therapist)

#### 3.3.5 Theme 5: HCP‐supported self‐management

While participants were generally positive about DHIs and identified where the tools could complement or improve usual care, only one individual stated that they would rather use a DHI than visit a HCP. Furthermore, there was little acknowledgement of the intervention being a self‐directed tool. There was a strong view amongst a subset of participants and HCPs that the DHI needs to be utilised alongside ongoing HCP support.

I suppose having the configurability that it can be driven by you and/or a medical professional where appropriate. (P9/FG3, Male‐40 years‐Epicondylitis)

Discussions focussed on how DHIs could facilitate conversations between patients and HCPs, especially in relation to goal setting. Participants believed that collaborative goal setting, which was recorded and monitored within a DHI, would offer accurate progress updates and facilitate discussions with HCPs about necessary programme amendments.

I think that would be really good, because then you can have a conversation about, if you haven't been doing it too much is there anything that, is it too difficult to fit in? or is it too painful? and then it opens a bit more of a conversation. (HCP07 Physiotherapist)

Discussions touched upon integrating the DHI within usual care, recognising that it may be unsuitable for some patients. It was understood that integration could be challenging to implement due to the current problems that people face getting an appointment to see HCPs.

I think there's a potential challenge there, but I think something where they feel able to feed in and know that you can look at it and potentially you can adjust their exercise programme, I think there's something quite beneficial about that. I think the thing with self‐management often […] typically with these patients, we'll see them and then say I'll see you in a month, go and do these exercises and the progressions I've given you and what a lot of patients, if I then speak to them, say, a month later, sometimes they'll say, look, I got a week in and it really flared the problem up but they haven't been that easily able to contact us to let us know or I haven't had availability to see them. (HCP01 Hand therapist)

### 3.4 Barriers to digital engagement

#### 3.4.1 Theme 1: Scepticism of digital management

Some participants were sceptical about digital management of DUL‐MSDs and were not satisfied that the same standards of care could be offered digitally. HCPs noted that some patients may be reluctant to engage with DHIs and could feel discouraged or ‘fobbed off’ when signposted to a digital programme instead of receiving usual care.

Some patients feel like when you signpost them to websites and apps and things they think that's you fobbing them off, that that's what this is, because the doctor didn't want to see you, the doctor doesn't want to treat you and they think oh, this app is just like, they don't realise it's doing the same things sometimes better than what we do. (HCP02 Rheumatologist)

Experiences of online physiotherapy during the Covid‐19 pandemic led some FG participants to express firm preferences for face‐to‐face physiotherapy over remote services. They believed that without an in‐person examination, physiotherapists would not be able to prescribe appropriate treatment. Reluctance to engage with DHIs stemmed from observing limitations of other online health information sources.

We went into lockdown, so were doing online physio sessions, but what good is that? They can't see or feel where the pain's coming from. (P3/FG1, Female‐51 years‐Hand/Thumb OA)

How specific can you be when you're being remote? You know, because somebody isn't sitting there looking at the size of your wrist and its shape and the way that it doesn't twist or, you know, to be able to advise properly. (P6/FG2, Female‐50 years‐Hand/Thumb OA)

#### 3.4.2 Theme 2: Challenges to implementing digital services

HCPs believed that a DHI could not replicate certain aspects of in‐person usual care. One aspect of usual care that was identified as a challenge to implement digitally was the level of individual tailoring that HCPs viewed as necessary to help patients optimise their recovery, while considering their work and personal circumstances. One HCP provided an example of working with labourers to regain their ability to perform fitness‐based activities while trying to mediate potential occupational setbacks. Other HCPs were concerned about the inability of DHIs to replicate the role and time commitment dedicated to checking patients' techniques and positioning, so that patients gained optimal benefits from exercise without aggravating their symptoms.

You can't always check how well people are doing it virtually. (HCP03 Occupational Therapist)

Several off‐putting limitations with DHIs were raised. One participant referred to DHIs removing the in‐person human empathy aspect of usual care, while another mentioned the difficulty of using DHIs for symptom checking.

You don't get empathy from a screen. (P2/FG1, Female‐55 years‐Tendinitis/Tenosynovitis)

#### 3.4.3 Theme 3: Concerns about incorrect treatment

Concerns were raised by participants with DUL‐MSDs about the consequences of following an unsuitable digital exercise programme. Reluctance to engage with a digital exercise programme was conflated by a combination of factors including previous misdiagnosis of their DUL‐MSD and concern around exacerbating painful symptoms due to a fear that pain equals damage.

I think three of us sound like we had a bit of a mis sort of diagnosis at the beginning, so there would be that danger if it was all online you could start following a programme that could potentially not do […] you've got to make sure that you're following the right diagnosis aren't you initially because in case you're doing some damage by following the wrong programme? (P5/FG2, Female‐54 years‐Epicondylitis)

Fear of making their symptoms worse or causing further damage was a key point discussed throughout interviews and FGs as a reason for avoiding aggravating activities, to the point where some participants reported doing very little to self‐manage their condition. This may influence engagement with any physiotherapy‐based treatment, whether in‐person or remote. Some HCPs expressed concerns about patients not performing the exercises correctly without someone to demonstrate and address any issues.

#### 3.4.4 Theme 4: No HCP involvement

Some participants with DUL‐MSDs were uncertain about the purpose of a self‐directed intervention that was not integrated within usual care, viewing it as pointless. For some, HCP input played a crucial role in their motivation to engage; without HCP involvement, they would be discouraged from using DHIs. Others expressed that they did not understand the purpose of engaging with a DHI if their metrics and progress were not reviewed by an HCP.

I think the hard thing is like if you're not supported by say a professional who's going to continue to see you well no one's going to see me anyway, what's the point I think is the, would be how it could be seen, whereas if, if you had something that was kind of progressing, like maybe there was reviews by somebody or said you know you do this programme for three months and then your GP gets a notification that you've like you've completed three months, so kind of check in with you, then I think that would be more motivating because you know you're not just left to your own devices sort of thing. (P13/Interview, Female‐32 years‐Tendinitis/Tenosynovitis)

It was considered important that HCPs were aware of their patients' progress and not having HCP involvement may be a barrier to digital health engagement.

Table 3 maps information about the barriers and facilitators to engagement with a DHI to the COM‐B model^31^ and presents solutions that could address these issues and achieve the target behaviour, which is to engage with a DHI. The COM‐B model helped to identify the barriers to behaviour, enabling solutions that will influence motivation, capability and opportunity.

**Table 3:** Barriers (B) and facilitators (F) to engagement with a digital health intervention for self‐management of distal upper limb musculoskeletal disorders and potential solutions and associated COM‐B domain.^42^

B/F to the target behaviour and COM‐B domain	Summary of evidence from focus groups and interviews	Intervention design feature(s) that could address the barrier/facilitator
B1. Lack of knowledge about self‐management of DUL‐MSD Knowledge Psychological capability Reflective motivation	While some people are proactive in searching for information about their condition and how to self‐manage it, others are not sure which websites to go to for this information and are more reliant on obtaining their information from an HCP. If HCPs did not have sufficient time to provide information, some people could have little knowledge of how to manage their condition. Additionally, being previously misdiagnosed means that some people are very uncertain about what information to access.	Resources where users are provided with information about how to self‐manage their specific condition with regard to exercise, devices, splints/supports and pain relief.
	Several HCPs mentioned pacing and joint education as concepts where finding the right approach to explaining the concept could be challenging. For example, it could be difficult to explain the importance of joint protection and scaling back on exercise when the patients' occupation involved heavy manual work.	Guidance on pacing and joint education. Resources about how to manage their condition at work.
B2. Limited experience of using digital interventions Physical opportunity Physical capability Psychological capability	Participants with DUL‐MSDs were confident in some capacity with using the internet and many had used smartphone apps or websites before. While they may have some expectation of how to use a digital platform to manage their DUL‐MSD, other less confident internet users will require a simple, accessible platform that is easy to use and contains clear instructions that allow them to navigate the platform.	Making the DHI easy to use with accessibility features, simple navigation and clear instructions.
	People with DUL‐MSDs wanted to ensure that they received the optimum benefit from using the digital platform. This was especially important when considering people who were not comfortable with using technology.	Introductory section—showing people how to use the various sections of the DHI, making sure that it is suitable for those with no experience of using digital tools.
B3. Challenges to using digital technologies Psychological capability Reflective motivation	Participants wanted reassurance that the information presented within the DHI is evidence‐based, credible and reliable. HCPs were aware that the amount of information available can be overwhelming, so having one resource where patients can go for their information and know it is reliable is important.	Indication that the DHI is credible and reliable.
	HCPs thought that it was important to keep users engaged with a digital platform. As such, it is important to provide features and content that keep users motivated to use the platform. This is especially important when users do not feel like they are progressing. People with DUL‐MSDs had found apps useful where they could look back and see how they had progressed and see logged data to compare with how they physically felt.	Highlight the benefits of the DHI. Provide reassurance of progression through being able to review exercise sessions and pain levels.
	People with DUL‐MSDs were more likely to engage with a digital intervention if it was free.	Emphasise that the DHI is free to use.
B4. Concerns about authenticity/credibility of digital intervention. Reflective motivation	People with DUL‐MSDs were keen for the digital platform to be endorsed by reputable organisations. The NHS was considered the most reputable, but other participants referenced the arthritis charity, vs. arthritis.	Endorsement by reputable organisations (NHS, vs. arthritis).
	Some people with DUL‐MSDs question the information that they read online unless they are signposted to it by an HCP. They want to know the source of the information and whether the source is reliable.	Clarity that information comes from reputable sources such as the NHS and vs. arthritis.
B5. Concerns about specific digital features Reflective motivation	There were concerns that the DHI could not replicate the in‐person aspects of usual care, such as checking a patient's technique when exercising.	Short videos and animations for the exercise programme, to see a demonstration of the exercises. However, the DHI cannot check the technique beyond demonstration of what the exercise should look like.
	Choosing the intensity of exercises was important to some people with DUL‐MSDs; they wanted to be able to choose their exercises dependent on their abilities and pain levels, rather than be preassigned exercises that never changed.	Feature optionality—e.g., choosing the intensity of the exercise programme.
F1. Immediate accessibility/ease of use Physical capability Psychological capability	Having readily available information was considered important, especially by HCPs, who viewed an accessible digital platform as something that could potentially save clinical time. People with DUL‐MSDs wanted something that they could start using immediately to help manage their condition.	Digital features of the DHI must enable accessibility.
F2. Ability to track and monitor data Reflective motivation	People with DUL‐MSDs had found apps useful where they could look back and see how far they had come and see logged data to compare with how they physically felt. Seeing improvements or drops in progress was useful to participants.	The DHI should have the ability to log data for later review, such as workouts, pain levels, goals, etc.
F3. Personalisation of intervention Physical opportunity	People with DUL‐MSDs wanted to customise their user experience, which meant using the digital platform in the way they preferred, personalising features and receiving notification in their preferred format. People wanted to have their treatment tailored to themselves and their individual needs.	Feature optionality ‐ goal setting and receiving notifications in their preferred format (i.e., by email or text message).
F4. Tailoring of intervention (tailored advice based on symptoms; levels of difficulty) Physical opportunity	Having information specific to their DUL‐MSD was one of the most important things to people living with DUL‐MSD conditions. They did not want to be overburdened with information that is not specific to them.	The DHI should assign users to a specific user experience based on their condition; this allows them to only see information relevant to their DUL‐MSD. Feature optionality—addition of exercises if users progress well through the exercise programme and indicate no issues with pain.
F5. Having access to gadgets/support devices and splints Physical opportunity	People with DUL‐MSDs wanted information and recommendations for gadgets and devices that would assist them in their daily activities, particularly around the home. People with DUL‐MSDs also wanted recommendations from their HCPs about what kind of splint or support they should wear for their DUL‐MSD. Due to disparities in the availability of NHS resources, HCPs did not always have splints and supports that they could show or provide to their patients.	The DHI will provide information on evidence‐based devices, gadgets and splints that are seen to help people with certain DUL‐MSDs. The DHI will also signpost to appropriate equipment/devices, which may help support self‐management.
F6. Input from a healthcare professional Automatic motivation Psychological capability	Some people with DUL‐MSDs and HCPs thought that the DHI should complement usual care and saw the intervention as a resource that would facilitate discussions between HCPs and people with DUL‐MSDs. People with DUL‐MSDs were motivated to use a digital platform where they would also get input from their HCPs on their progress and treatment plan.	N/A—the DHI cannot directly address this facilitator at this time as this is a self‐management digital intervention.
F7. Social support/forum Social opportunity	Some people with DUL‐MSDs thought that having some form of support group would be valuable for people with DUL‐MSDs. A forum was one form of social support group that was briefly discussed.	Explanation that person could engage with family members/friends; signposting to support resources. The DHI is not able to provide a forum through the digital platform.

Abbreviations: COM‐B, capabilities, opportunities, motivation behavior; DHI, digital health intervention; DUL‐MSD, distal upper limb musculoskeletal disorder; HCP, healthcare professional; NHS, National Health Service.

## 4 DISCUSSION

This study provides valuable insights from both people with DUL‐MSDs and HCPs into potential barriers and facilitators to engagement with a DHI, which need to be considered during development of any such intervention.

Treatment and management of people living with a painful DUL‐MSD are challenging. Research should develop novel approaches to support self‐management and personalise, improve and optimise care in alignment with the NHS's long‐term plan.^47^

Barriers and facilitators to engagement with a DHI were based on participants' individual preferences and previous experience of using digital health tools or services, which was heightened during the Covid‐19 pandemic. These findings highlight the importance of preferences, personalisation, tailored content, usability and HCP‐supported self‐management when developing a DHI. Participants, particularly HCPs, considered how a digital self‐management rehabilitation programme could replicate and be integrated into usual care.

A combination of condition‐specific tailoring, choice of exercise intensity, user‐centred design and personalisation of content was important and valuable to people with DUL‐MSDs. There was a need for information, guidance and an exercise programme specific to their condition, capabilities, pain levels and preferences. These findings are reflected in evidence from a review evaluating barriers and facilitators to engaging with DHIs for lower back pain, which reported that personalisation to user experience was valued. The review also reported that symptom‐specific tailoring was thought to enhance engagement, improve intervention effectiveness and minimise self‐management burden, even though this tailoring had not been included within interventions.^48^

While condition‐specific information was valued, people with DUL‐MSDs were clear that too much generic information was burdensome and off‐putting. Work that addressed barriers and facilitators of health and lifestyle apps emphasised that presenting information in a concise and understandable way was preferred as it reduced the time and effort required to use apps.^49^, ^50^ Other research reported that too much information impacted negatively on understanding, engagement and motivation to use DHIs.^48^ Developers of DHIs should consider how much information is presented and ensure that information is relevant.

Goal setting was understood to be a key element of personalisation. However, people with DUL‐MSDs and HCPs had differing views on its value. HCPs discussed the benefits of goal setting, while one individual with a DUL‐MSD discussed avoidance of goal setting due to the frustration experienced if they did not achieve their goal. In this study, people with DUL‐MSDs were more enthusiastic about tracking and monitoring progress. Previous research cited goal setting and tracking progress as effective motivators.^50^, ^51^, ^52^ However, for optimal engagement and personalisation, DHIs would go beyond tracking data and provide interpretation in the form of individual feedback and advice.^53^, ^54^

Usability was an important facilitator of engagement with a DHI. Previous research presented ease of use as a factor associated with perceived usefulness of lifestyle apps, both of which were significantly associated with user retention.^55^ Participants in the current study rated themselves as fairly or very confident internet users, a characteristic associated with access to and engagement with DHIs.^56^ Digital literacy was discussed among FG participants, emphasising the importance of an accessible, intuitive and simple digital tool for less confident internet users. Evidence from a systematic review of barriers and facilitators for the utilisation of lower back pain DHIs corroborated that participants value and expect information to be easily accessible, understandable and structured. The review highlighted multiple factors to consider when planning DHIs, including user‐friendliness and ease of registration and logging in.^48^

Other usability aspects identified in this study included credibility and trustworthiness. Participants considered a DHI to have enhanced usability if information was verified and endorsed by reputable organisations, with the NHS perceived as the most credible. Similar findings were reported by other studies, where clinical endorsement was important to users as it improved perceived content quality.^57^, ^58^

Within the current study, HCP‐supported self‐management was both a facilitator and a barrier to using DHIs; HCP input and support was a facilitator, while the absence of HCPs discouraged some from using a DHI. This illustrates a preference for a combination of digital and HCP‐supported self‐management. This corresponds with evidence from a systematic review of DHIs for lower back pain, where users expressed that they wanted direct support from HCPs and that this would enhance engagement.^48^ Additionally, an evaluation of an online‐based physical activity behaviour change programme reported that many participants preferred face‐to‐face sessions,^49^ indicating that online services are not suitable for everyone. The finding of HCP support as a facilitator of DHI use implies that some participants with DUL‐MSDs require additional support. However, the DHI that will be developed as part of the current project is intended to be a self‐management tool to empower people to manage their DUL‐MSD independently. As such, it is important that the DHI provides people with the knowledge and skills to feel confident to actively self‐manage their DUL‐MSD.

Participants were keen for the DHI to facilitate conversations and support from their HCP. HCPs perceived the DHI as a complementary tool to usual care rather than a stand‐alone programme. Evidence from a systematic review indicated that user benefits of DHIs for lower back pain included increased understanding of their condition and better communication with HCPs during consultations.^48^ The review concluded that DHIs should be designed to help facilitate efficient and helpful discussions.

## 5 LIMITATIONS

This study had several limitations. While purposive sampling was used,^33^, ^34^ some of the defined criteria were difficult to achieve when recruiting people with a DUL‐MSD; therefore, a more pragmatic sampling approach had to be taken. This meant that our sample of participants with DUL‐MSDs was overrepresented by females, white British ethnicity and those who were fairly or very confident in their ability to use the internet. Consequently, issues relating to digital literacy and IT accessibility were not able to be fully explored and will be a key focus during the development and testing phases of this programme of research. There were more people with hand/thumb OA in our sample due to it being a more prevalent condition.^3^

Although the study had a wide recruitment strategy, most of the recruitment was through social media, community and charitable organisations. This recruitment strategy may have resulted in a self‐selecting sample of engaged participants with an interest in digital health and improving their DUL‐MSD.

## 6 IMPLICATIONS FOR PRACTICE AND FUTURE RESEARCH

This study's findings will inform the development of a DHI for people living with DUL‐MSDs. While the intervention must be user‐friendly, accessible and empower people to self‐manage their condition, it must be acknowledged that digital rehabilitation self‐management is not for everyone.

The DHI and evidence base will require continual evaluation and frequent updating to ensure that content reflects current medical knowledge and information about how to manage DUL‐MSDs. Future research needs to investigate barriers and facilitators from a more diverse sample, including less confident internet users.

## 7 CONCLUSION

DHIs contribute towards providing supported self‐management and personalised care. Before developing a DHI, the barriers and facilitators that impact user engagement are important to identify. A variety of barriers (scepticism of online management, challenges to implementing digital services and concerns about incorrect treatment) and facilitators (digital design features, personalisation, tailoring and usability) were identified and will support the development of a DHI for managing DUL‐MSDs that is relevant, acceptable and supportive. As some participants discussed requiring HCP input, there is a need to ensure that people are empowered to participate in their own care and a DHI may provide this.

## AUTHOR CONTRIBUTIONS

**Samantha J. Mason**: Writing—original draft; writing—review and editing; formal analysis; data curation; visualisation. **Lucy M. Brading**: Data curation; writing—review and editing; investigation; formal analysis. **Kathleen Kane**: Data curation; writing—review and editing; investigation; formal analysis. **Philip G. Conaghan**: Funding acquisition; writing—review and editing. **Sarah R. Kingsbury**: Funding acquisition; writing—review and editing. **Gretl A. McHugh**: Funding acquisition; conceptualisation; writing—original draft; writing—review and editing; methodology; formal analysis.

## CONFLICT OF INTEREST STATEMENT

The authors declare no conflict of interest.

## ETHICS STATEMENT

Ethical approval for this study was obtained from West Midlands—Edgbaston Research Ethics Committee (21/WM/0277). All participants provided electronic informed consent before taking part in the study.

## Supporting information

Supporting information.

## Data Availability

The data that support the findings of this study are available on request from the corresponding author. The data are not publicly available due to privacy or ethical restrictions.
