# Supplementary material for: Barriers and facilitators to engaging with a digital self‐management programme for painful distal upper limb musculoskeletal disorders: A qualitative exploratory study
Source: Health Expect. 2024 Jun 10;27(3):e14056. doi: 10.1111/hex.14056 (PMC11164711; doi:10.1111/hex.14056)
Supplement: Supplementary file 1 — Supporting information. [file HEX-27-e14056-s001.docx]

**Supplementary File 1: Examples of the trigger materials used in interviews and focus groups to illustrate features of the Digital Health Intervention**

**Pain tracker**

Add daily reflection

How is your pain right now?

**MANAGING MY PAIN**

**+**

**+**

**6**

Worst pain

imaginable

No pain

at all

**Version 1.1 | 17th May 2022**

**D-MAPP trigger materials | Page 5**

**MONITORING MY PAIN**

Where does it hurt?

Location

What besides pain are you feeling?

Associated symptoms

Did you take medication for the pain?

Medications

**Version 1.1 | 17th May 2022**


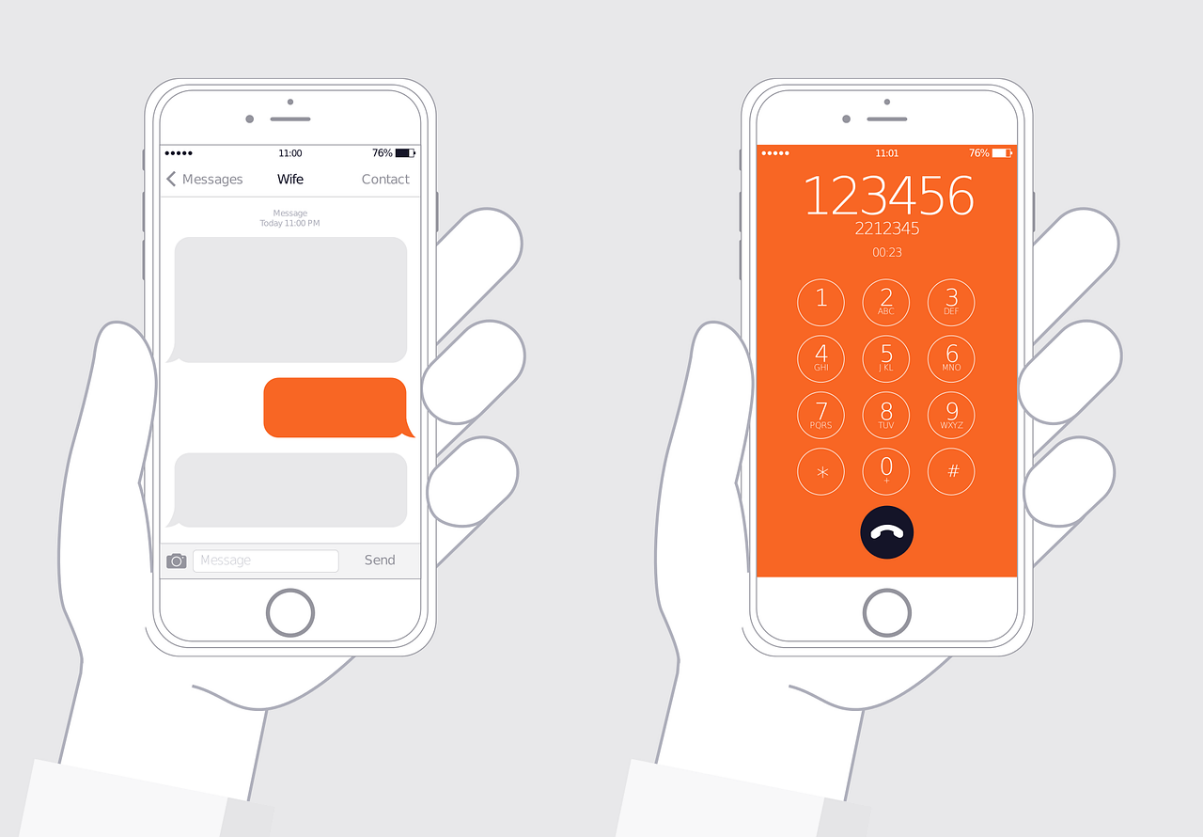

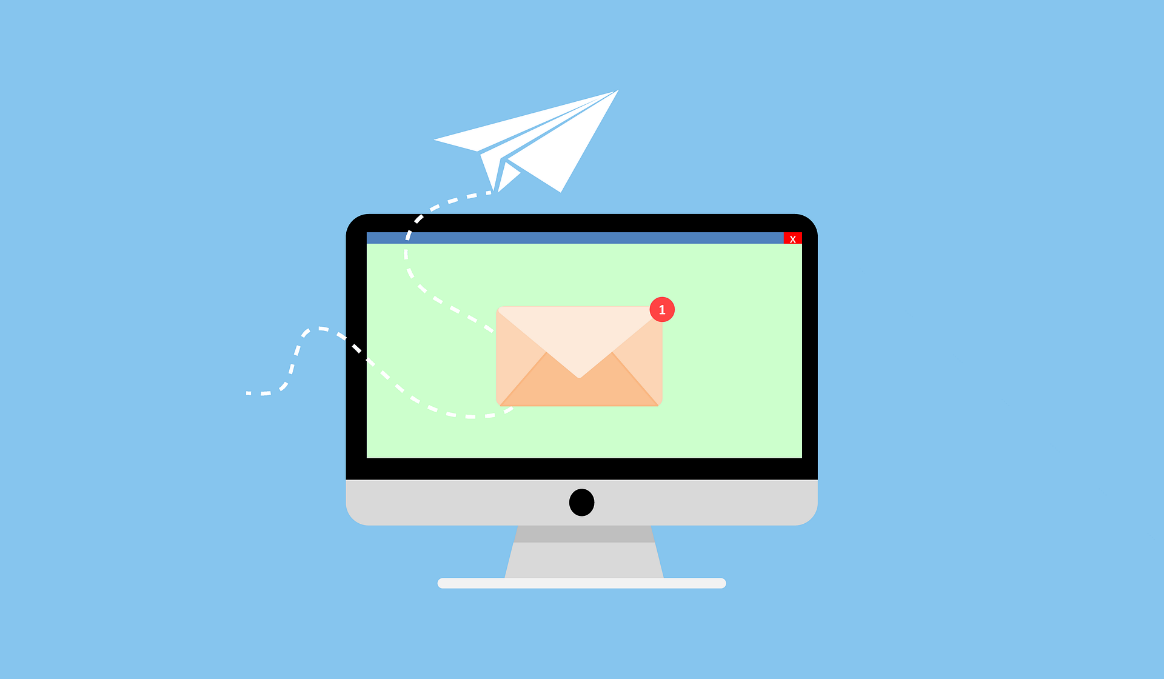

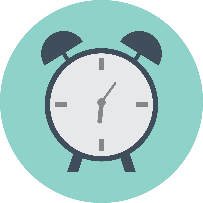


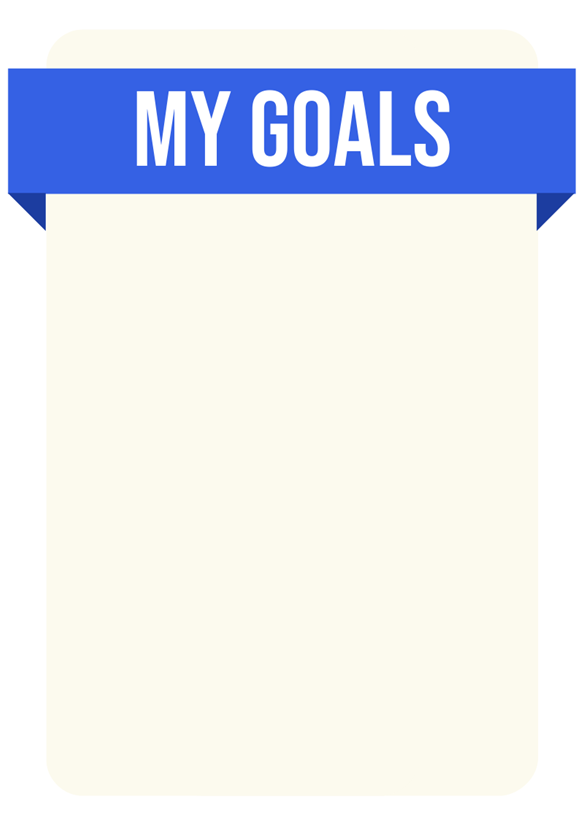


I will repeat this goal...

I will...

For...

5 min

10 min

15 min


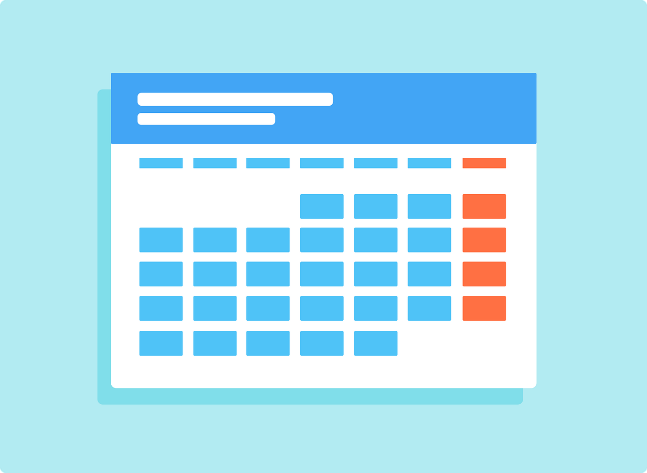


do hand stretches

Remind me at this time...

**MM: HH**

do hand stretches

**Version 1.1 | 17th May 2022**

**Reminders**
